# The Role of Surgeon Gender in Patient Surgeon-Selection in Plastic and Reconstructive Surgery: A Crowdsourcing Analysis

**DOI:** 10.1093/asjof/ojae007.092

**Published:** 2024-04-12

**Authors:** Helen Xun, Maria J Escobar-Domingo, Jose Foppiani, James Fanning, Angelica Hernandez Alvarez, Ashley Boustany, Bernard Lee

**Affiliations:** BIDMC, Boston, MA; Beth Israel Deaconess Medical Center, Harvard Medical School, Boston, MA; Beth Israel Deaconess Medical Center, Harvard Medical School, Boston, MA; Beth Israel Deaconess Medical Center, Harvard Medical School, Boston, MA; Beth Israel Deaconess Medical Center, Harvard Medical School, Boston, MA; Beth Israel Deaconess Medical Center, Harvard Medical School, Boston, MA; Beth Israel Deaconess Medical Center, Harvard Medical School, Boston, MA

## Abstract

**Goals/Purpose:**

Plastic and reconstructive surgery (PRS), and especially aesthetic surgery, is uniquely characterized by an increased ability to research and select their surgeon. As the increasing number of female plastic surgeons begin to establish themselves in practice, it remains understudied if gender or implicit gender biases is a variable in patient surgeon-selection.

Wallis, et al.’s landmark paper in 2023 suggested that female surgeons exhibited a lower risk-adjusted likelihood of adverse postoperative outcomes, including mortality, hospital readmission, and major medical complications, although the precise mechanisms remain unclear. Currently, it is unknown if the public is aware of these findings, and if it these findings would impact patient-surgeon selection.

Patient-surgeon selection may be an opportunity where PRS may lead surgical subspecialties in addressing implicit surgeon gender biases due to the predominance of female patients, and improved gender representation in surgical training programs compared to other surgical subspecialties. The purpose of this study is to explore public preferences and perspectives on the role of surgeon gender in patient surgeon-selection in PRS, and if knowledge of gender-correlated surgical outcomes impact surgeon selection. A secondary aim is to examine if these public preferences differ between reconstructive versus aesthetic surgery.

**Methods/Technique:**

A cross-sectional survey was distributed through Amazon’s Mechanical Turk (mTurk). Subjects were at least 18 years old and residents of the United States. Participants were briefed about hypothetical scenarios involving aesthetic and reconstructive surgical procedures, and then asked to choose either a male and female surgeon. They were then presented with an excerpt of Wallis, et al. (2023) findings, and reassessed for surgeon gender preference. The participant cohort was stratified by gender, classifying them as female and male responders. Differences between groups were evaluated using the unpaired t-test and Fisher exact tests. Multivariate logistic regression models were constructed to assess associations between plastic surgeon gender preference and sociodemographic characteristics. Stata statistical software (STATA Corp., BE, 18.0) was used to conduct all statistical analyses.

**Results/Complications:**

A total of 547 participants were included. Of those, 310 (56.7%) were male and 237 (43.3%) were female. The mean age was 33.8 (SD 6.8) for the male group and 35.1 (SD 7.2) for the female group. Both groups had similar percentages of level of education (p=0.071). Significant differences were observed in race (p=0.004), ethnicity (p<0.001), U.S. region of residency (p<0.001), and religion (p<0.001) (Table 1). Both groups had similar percentages of previous PRS background/interest (M: 81.3%, F: 86.1%, p=0.164). However, female participants had higher percentages of aesthetic background/interest (86.1%) compared to the male counterparts (78.7%, p=0.033). Similar interest/background in reconstructive procedures was found between groups (M: 60.0%, F: 45.6%, p=0.222) (Table 2).

Statistically significant differences emerged in overall preferences for physician's gender (p<0.001), as well as preferences for the gender of plastic surgeons conducting aesthetic non-surgical (p<0.001), aesthetic surgical (p<0.001), and reconstructive surgical procedures (p<0.001), particularly favoring the same-gender preference. A higher percentage of male participants believed that the plastic surgeon's gender impacts surgical outcomes (77.4% vs. 62.0%, p<0.001), associating better outcomes with male surgeons (91.2%). The majority of female responders favored female surgeons for better outcomes (63.9%, p<0.001). Following exposure to an excerpt from the Wallis, et al. (2023) published article, preferences for female plastic surgeons increased among both groups, with statistically higher rates in the male group (79.4% vs. 69.6%; p=0.010) (Table 3).

Multivariate logistic regression analysis demonstrated that for aesthetic surgical procedures, female gender [10.65 OR; 95% CI (5.65–1.29); p<0.001], living in the Southwest region [1.88 OR; 95% CI (1.11–12.62); p=0.033], were factors independently linked to a higher preference for female plastic surgeons. Conversely, age [0.97 OR; 95% CI (0.94–0.99); p=0.025], and having a background/interest in reconstructive procedures [0.57 OR; 95% CI (0.35–0.93); p=0.027] were associated with a lower preference for female plastic surgeons in this category (Table 5).

Following presentation of an excerpt from the Wallis, et al. (2023) article, Hispanic ethnicity [2.67 OR; 95% CI (1.31–5.48); p=0.007], residing in the Southeast region [3.12 OR; 95% CI (1.43–6.81); p=0.004], having a background/interest in aesthetic [2.05 OR; 95 %CI (1.16–3.63); p=0.014], and reconstructive procedures [3.86 OR; 95% CI (2.29–6.51); p<0.001] were associated with a higher likelihood of shifting preference towards female plastic surgeons (Table 7).

**Conclusion:**

Participants demonstrated a predominant preference for surgeons of the same gender across aesthetic non-surgical, aesthetic surgical, and reconstructive procedures. Initially, gender biases affected perceptions of surgeon gender and surgical outcomes, particularly notable among male participants. However, this preference notably changed following exposure to a segment of published literature on surgeon outcomes based on gender. These findings support the importance of gender diversity in plastic surgery due to patient preference, and suggests that implicit gender biases in patient surgeon-selection persist, but can potentially be addressed with patient education. Rigorous future studies are required to expand hypothesizes generated from this proof-of-concept study, and should emphasize including non-binary populations.

